# Chicago Medical Society

**Published:** 1881-05

**Authors:** 


### ARTICLE IX.

### CHICAGO MEDICAL SOCIETY.

Stated Meeting, April 18, 1881. Dr. E. Ingals, President, in the chair.

The three new members proposed at the last meeting were elected.

#### VACCINATION AND VACCINE VIRUS.

Dr. N. S. Davis related three cases in nursing children, who, not being susceptible of vaccination, had been exposed to small-pox without taking the disease; and made the inquiry: "Should nursing infants under two or three months of age be vaccinated?" He also inquired whether bovine virus was a better protective than humanized virus?

Dr. W. E. Clark said he generally declined to vaccinate infants because he had once had a case of adenitis, and had seen other bad results.

Dr. O. C. DeWolf, Health Officer, said he had young children vaccinated with a good result. Of several cases which had been afterward taken to the Small-Pox Hospital with their mothers, not one had contracted the disease. As to the greater immunity conferred by bovine virus, it was doubtful. Dr. Jones did not use bovine virus because very young children were less susceptible to it, and because the arm was made more sore by it. Dr. DeWolf related that a mother had been taken with variola a few days ago who nursed a baby two days old. It was not vaccinated, and was fourteen days old to-day, the time when the eruption should have appeared; but the infant has just died in convulsions, probably being overwhelmed with small-pox, although no symptom manifested itself before this. Had the child been vaccinated, it would have lived. He had never seen a fatal case in one who had been vaccinated. The Health Department employed bovine virus exclusively, because more readily obtained. The disease was not at all modified by old vaccinations, some had confluent small-pox who had many cicatrices on their arms. Eighty-nine per cent. of the cases of secondary vaccination were successful, and he never saw a case of small-pox among them in four years.

Dr. John Bartlett said that children who were not susceptible of vaccination might take small-pox, he had observed six different cases of that kind, two of them in the practice of Dr. Byford.

Dr. E. Ingals believed the rule to be that a case which resisted vaccination, did not easily take small-pox. He had been vaccinated many times, only the first successfully, and he was subsequently exposed to small-pox a great deal, but finally took varioloid from a case of confluent small-pox. He inquired whether a repetition of vaccination within a few months would take?

Dr. R. S. Hall had seen a successful case of that kind, and had no doubt that if we should re-vaccinate some weeks after the first vaccination had taken, many cases would prove successful.

Dr. Jane E. Walton re-vaccinated a child after six days, and both vaccinations were successful.

Dr. N. S. Davis said that immediate re-vaccination was the only test of a good vaccination and should be tried; the great majority of cases were not susceptible to it. He thought the time for compulsory re-vaccination might be fixed between the age of sixteen and twenty-one, and he thought the majority would be safe for a life-time. But he mentioned that some diseases like typhoid, or some change of climate, might interfere with the immunity conferred by vaccination. He had always used humanized virus until a year ago. He had never seen any bad results follow.

The Society now drew up resolutions on the occasion of the death of Dr. Mills Olcott Heydock, M.D., which happened on April 17. Dr. Heydock had at one time occupied the Chair of Materia Medica in the Chicago Medical Callege.

Dr. DeLaskie Miller was elected a delegate to the International Medical Congress, and the Society adjourned.

---

The West Chicago Medical Society has not held a meeting for two months. The immediate cause of it is a want of attendance on the part of members.

